# Effect of active and passive distraction techniques while administering local anaesthesia on the dental anxiety, behaviour and pain levels of children: a randomised controlled trial

**DOI:** 10.1007/s40368-022-00698-7

**Published:** 2022-03-10

**Authors:** S. Shekhar, B. S. Suprabha, R. Shenoy, Ashwin Rao, Arathi Rao

**Affiliations:** 1 Department of Pediatric and Preventive Dentistry, Manipal College of Dental Sciences, Mangalore, Manipal Academy of Higher Education, Manipal, India; 2 Department of Pediatric and Preventive Dentistry, Manipal College of Dental Sciences, Mangalore, Light House Hill Road, Mangalore, Karnataka 575 001 India; 3 Department of Public Health Dentistry, Manipal College of Dental Sciences, Mangalore, Manipal Academy of Higher Education, Manipal, India

**Keywords:** Audio-visual, Play therapy, Distraction, Nerve block, Dental care

## Abstract

**Purpose:**

The aim of the study was to compare the effect of a stress ball, an active distraction technique with audio-visual eyeglasses, a passive distraction technique during local anaesthesia administration, on dental anxiety (primary outcome), behaviour and pain levels of children (secondary outcomes).

**Methods:**

In this randomised controlled parallel arm trial involving 123 children aged 8–12 years, who required dental treatment under inferior alveolar nerve block, children were randomly allocated into the following three groups: Group 1: Stress ball, Group 2: Audio-visual eyeglasses, Group 3: Control group (basic behaviour guidance without distraction). Dental anxiety was measured using modified child dental anxiety scale and pulse rate, behaviour was rated using Venham’s scale and pain was measured by both self-reporting and observational scales.

**Results:**

No significant difference between the groups was observed for dental anxiety, but a significant decrease was seen in dental anxiety scores within all groups. No significant differences were seen between the groups for behaviour ratings and pain scores.

**Conclusion:**

Use of active stress ball distraction or passive audio-visual eyeglasses during local anaesthesia administration decreased dental anxiety but did not result in a significant improvement in the dental anxiety, behaviour and pain levels when compared to basic behaviour guidance without distraction.

**Clinical trial registration:**

The clinical trial was registered at Clinical Trials Registry–India (CTRI Reg no: CTRI/2019/04/018768, Dated 24 April 2019).

**Supplementary Information:**

The online version contains supplementary material available at 10.1007/s40368-022-00698-7.

## Introduction

The use of local anaesthesia is one of the most common practices of pain control in paediatric dentistry. However, it is commonly associated with pain and increased anxiety levels in children (Ram and Peretz 2002). This results in a negative attitude towards dental care despite the effective action of local anaesthesia (Ram et al. 2010). Anxiety is a response to imminent danger expressed through a combination of biochemical alterations and influenced by memory, personal history and social context (Corah et al. 1978). Dental anxiety is a common issue faced by the people of all ages, though it seems to be more common in children and adolescents (Mendoza-Mendoza et al. 2015). This can cause behaviour management problems for the dentist, leading to unpleasant experiences for both the dentist and the child (Armfield and Heaton 2013).

Distraction is a commonly used non-pharmacologic behaviour guidance technique to decrease procedural pain. The technique shifts the child's focus to something engaging and attractive; his or her capacity to attend to painful stimuli is hindered, thereby reducing pain and anxiety (Koller and Goldman 2012; Barreiros et al. 2018). Distraction techniques are of the following two types: active and passive. Active forms of distraction promote the child's engagement in an activity during the procedure therefore, tend to involve several sensory components. In the passive form, distraction is achieved through the child’s observation of a stimulus rather than his/her active participation (Koller and Goldman 2012). The most frequently used method of passive distraction is the use of audio-visual aids (Barreiros et al. 2018). Audio-visual (AV) eyeglasses are lightweight, goggle-like, portable set of glasses with a head mounted display and earphones that connect to devices such as television, mobile phones, for private viewing by the child (Chaturvedi et al. 2016). They engage the visual and hearing sensations of the child and partially isolate the child from the dental environment (Ram et al.^2010^).

Play therapy involves the use of play materials by the therapist to resolve psychological issues like anxiety. It uses the concept of symbolic play along with age-appropriate language skills (Hall et al. 2002). Play therapy in the form of a stress ball or a soft rubber ball that changes shape and colour when pressed has been utilised as an active distraction method in managing pain during paediatric operative procedures such as phlebotomy and catheter insertion. They work on the concept of reducing attention to pain by competing with the sensory stimuli of pain. They are soft and fun to press for children and a low-cost form of active distraction (Sadeghi et al. 2013; Aydin et al. 2016). Although this method has been used in paediatrics, its effectiveness in reducing pain and dental anxiety and improving behaviour during local anaesthetic injection for dental treatment in children is yet to be studied.

Comparative studies between active and passive distraction methods during paediatric procedures conducted earlier have found mixed or inconclusive results (Koller and Goldman 2012). Active distraction techniques can be more effective because of multisensory engagement. However, the passive distraction requires minimal involvement of the child and hence has the advantage of not depending on the child’s skills to conduct the technique (Ram et al. 2010; Koller and Goldman 2012). Considering that the stress balls are easy to use and do not require complex skills (Sadeghi et al. 2013), there is a need to study the effect of this technique in comparison with commonly used passive distraction techniques such as audio-visual distraction, in decreasing pain and anxiety and improving cooperation of the child during dental treatment. Thus, our study aimed to evaluate and compare the effect of a stress ball (active) distraction technique with AV eyeglass distraction (passive) technique during the administration of local anaesthesia (inferior alveolar nerve block), on dental anxiety, with basic behaviour guidance without distraction serving as control. In addition, the effect on behaviour and pain levels during the administration of local anaesthesia were assessed as secondary outcomes.

## Material and methods

### Study design

The investigation was designed as a randomised controlled parallel arm design, with a balanced allocation ratio of 1:1:1. The trial was registered at Clinical Trials Registry, India (Reg. no.: CTRI/2019/04/018768, dated 24 April 2019).

### Settings and duration

The study was conducted in the Department of Paediatric and Preventive Dentistry at a teaching dental hospital. The study commenced on 8 May 2019 and completed on 26 May 2021.

### Participants

The study sample was derived from the population of child patients who visited the paediatric dental department for dental treatment and the principal investigator enrolled the participants. Children aged 8–12 years, with positive (+) ratings of Wright’s modification of Frankl behaviour-rating scale (Stigers 2016) during the initial examination appointment in our dental clinic and who required extraction/pulp therapy/restoration for deep caries lesions in lower primary and first permanent molars under local anaesthesia (inferior alveolar nerve block) were included. The criteria for exclusion were the presence of any systemic and/or mental illness, dental treatment under local anaesthesia in the past 6–7 weeks (Rocha et al.^2009^).

### Ethical issues

Parents of the participants were informed about the intervention and a written informed consent was obtained. Written informed assent was obtained from the child participants. Participation was voluntary, and no compensation was provided to participate in the study. Participants were free to withdraw from the study at any point in time and were assured that their participation or non-participation will not interfere with their routine dental treatment. The study was conducted after clearance by the institutional ethics committee. (Protocol number: 18094, dated 13 October 2018). All procedures were performed in accordance with the ethical standards of the institutional and national research committee and the 2013 amendment of the 1964 Helsinki declaration.

### Intervention

The participants were randomly allocated into the following three groups:

#### Group 1: Stress ball (active distraction)

Patients were introduced to the stress ball made of a multi-coloured silicone material with a diameter of 5 cm. The patients were asked to play with it while the local anaesthetic block was being administered. The children were instructed to keep pressing the ball, focus on it and observe colours as a part of the play therapy (Aydin et al. 2016; Sadeghi et al. 2013), thereby engaging the child while the local anaesthetic solution was being administered.

#### Group 2: Audio-visual group (passive distraction)

**Fig. 1:**
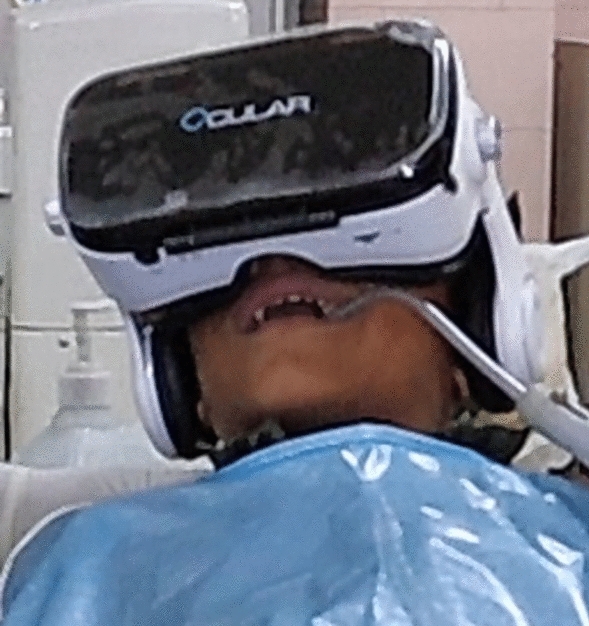
Illustration of AV eyeglasses used in Group 2

AV eyeglasses with built in headphones (Ocular Grand Fully Adjustable Headset with Inbuilt Headphones, Ocular VR, Aztek Hub LLP, Mohali, Punjab, India) and 3.5 MM jack compatible with mobile phone (One Plus 5 T, Midnight black, 128 GB, One Plus, Shenzhen, China) was used (Fig. 1). They are flexible, allowing adjustments to be made according to the patient’s morphology. They incorporate soft cushioning for the face and ventilation holes to dissipate heat from the smartphone. The lens is designed to reduce eye fatigue, eliminate the incidence of simulator sickness with a field view of 120°. The child was introduced to the AV system before the procedure and was given a choice of four age-appropriate cartoon movies with similar characteristics to be played for complete auditory and visual engagement.

#### Group 3: Control group (basic behaviour guidance techniques without distraction)

Communication (a standard set of verbal instructions based on a pre-script were given regarding local anaesthetic injection with age-appropriate euphemisms) with verbal positive reinforcement was used. Simple terms like “a slight pinch will be felt after which the tooth will fall asleep" were used to explain the procedure. The patients were informed that their “lips and tongue will feel numb after the medicine is given” and were instructed not to bite on them.

Communication and positive reinforcement were also used in Group 1 and 2, in a similar manner as Group 3.

#### Piloting

All interventions described in the three groups were piloted on five child patients each, prior to the onset of the study, to test for the feasibility of interventions, clarity of instructions base on a pre-script and as a part of training the principal investigator. Based on the observations during piloting, no changes were done to the pre-script.

#### Administration of the inferior alveolar nerve block

After drying with cotton gauze, a sterile cotton applicator tip was used to apply a topical anaesthetic gel containing 20% benzocaine (Precaine B, Pascal International, Bellevue, WA, USA) to the injection site. The applicator tip was inserted into the gel container and rotated three times. The gel was massaged onto the mucosa with moderate pressure for 30 s. After 3 minutes, the excess topical anaesthetic was removed using a sterile cotton gauze (Malamed 2004). The local anaesthetic nerve block was administered using a 27-gauge needle, with a length of 25 mm, using a syringe and cartridge system (Septodont Healthcare India Pvt. Ltd, Maharashtra, India) 0.1.5 ml of 2% lignocaine with 1:80,000 adrenaline was injected (Soxman and Malamed 2015).

The needle was kept out of direct view of the child during the administration of the anaesthetic. The cheek was stretched to make the tissue taut. A small amount of solution was injected, and after a negative aspirate, the needle was advanced until bony contact was made. The rate of administration was 1.5 ml of anaesthetic over a minimum of 60 s. The needle was slowly withdrawn and when approximately half the needle length remained, it was re-aspirated. If negative, a portion of the remaining solution was used to anaesthetise the lingual nerve. The needle was then withdrawn and made safe (Malamed 2004). Standardisation of the technique was achieved by the primary investigator, after performing the same procedure on at least ten patients under supervision, before the start of the study. A single operator did all procedures, in the presence of parents/guardians.

### Outcome measures

A questionnaire consisting of questions on the history of earlier dental visit (first visit or not), past dental and medical experiences of the child, socioeconomic status, parental anxiety and temperament of the child was administered to the accompanying parent prior to the procedure. The effects of these factors on the outcome measures were assessed as these factors are known to influence both dental anxiety and behaviour (Klingberg and Broberg 2007; Gustafsson et al. 2007; Suprabha et al. 2011; Assunção et al. 2013). The socioeconomic status was determined using the modified Kuppuswamy scale (Saleem 2020). The parental anxiety was measured using the modified Corah’s dental anxiety (MDAS) scale. The scale consists of five items, which is scored on a Likert scale with scores ranging from 5 to 25 (Humphris et al. 1995). The temperament of the child was measured using the EAS (Emotionality, Activity, Shyness) Temperament Survey for Children (parental ratings) consisting of 15 items, five corresponding to each of the three temperaments. Total scores thus ranged from 5–25 for each temperament type and a mean was calculated for each temperament (Emotionality, Activity and Shyness) (Boer and Westenberg 1994). Past dental and medical experiences were recorded using a structured closed-ended questionnaire.

Prior to administration of the local anaesthetic agent and introducing the child to the intervention, dental anxiety of the child was measured using the faces version of the Modified Child Anxiety Dental Scale (MCDAS_(f)_). The MCDAS_(f)_ scale is a reliable and valid indicator of dental anxiety among children. It uses simple language to cover the eight items or questions ranging from attending the dentist to extractions and general anaesthesia (Howard and Freeman 2007). Scores may range from 8 to 40, with scores below 19 indicating absence of state anxiety, scores higher than 19 indicating the presence of state anxiety (Aminabadi et al. 2012). The dental anxiety of the child was re-recorded soon after the administration of local anaesthetic agent. The variations in the pulse rate were recorded by another investigator before the intervention, during the injection of the local anaesthetic and 1 minute after the removal of the needle from the tissue, using a pulse oximeter (HHP-201, Monarch Meditech, Varachha, Surat, Gujarat, India), as an objective measure of dental anxiety.

Following the administration of local anaesthesia, each child was asked to rate the pain they felt during the injection using the Wong Baker Faces Pain Rating Scale (WBFPRS). The WBFPRS is a horizontal scale of six hand-drawn faces, scored from 0 to 10, ranging from a smiling, no hurt face on the left to a crying, hurts the worst face on the right (Garra et al. 2010). To overcome the drawback of the self-reported scale due to influence of the child’s cognitive ability and situational factors on the outcome, FLACC scale (Faces Legs Activity Cry and Consolability scale), that is assessed by an observer for five categories (faces, legs, activity, cry, consolablity) of the child’s behaviour, was used as an adjunct. Each category in FLACC scale is ranked on a three-point scale (0–2), resulting in a total score ranging from 0 to 10. Based on the total score of the five categories, the pain is categorised into the following four levels [Relaxed and comfortable (Score = 0), Mild discomfort (Score = 1–3), Moderate Pain (Score = 4–6), Severe discomfort/pain or both (Score = 7–10)] (Nilsson et al. 2008).

Assessment of child’s behaviour during the entire length of the procedure was done using the Venham’s behaviour rating scale that ranges from zero (signifying total cooperation) to five, (signifying general protest with no compliance or cooperation) (Venham et al. 1980). The peak score was recorded for the most non-collaborative child behaviour observed in response to the local anaesthetic injection procedure (Cademartori et al. 2017).

Scoring for behaviour and pain was done by observers after video recording the procedure for better reproducibility than real time assessment. The recording period started when the operator started applying topical anaesthetic and ended when the needle was removed from the tissue. The video camera was hidden and mounted at a distance from the child's direct vision, such that the child's entire body was visible in the video recording.

Assessment of the behaviour and pain was done by two examiners. Intra-examiner and inter-examiner reliability was assessed using Cohen’s Kappa statistics. Reliability checks were done using 10% of recordings. Inter-examiner reproducibility for both FLACC and Venham’s criteria was 0.783, depicting substantial agreement. The intra-examiner reliability value was one, depicting a total agreement for FLACC scale, for both the examiners. For the Venham’s behaviour rating scale, intra-examiner reliability was one and 0.783 for examiner 1 and 2, respectively.

#### Sample size

The sample size was calculated assuming a mean difference of 5.1 between the groups in MCDAS_(f)_ as follows scores for dental anxiety (primary outcome measure), with pooled standard deviation of 7.2 and an effect size of 0.7, based on earlier studies (Barreiros et al. 2018), at 95% level of significance and 80% power of the study. It was 41 for each group, thus making it 123. The sample size was calculated using G*Power 3.1 software.

### Method of randomisation

Randomisation was done by a statistician who was otherwise not part of the intervention. Block randomisation using a varied block size of 6 and 12 was done. The block sequences (ABCBAC, BACBAC, ACBBCA etc.) were computer generated, followed by a random allocation of the samples to the blocks using a random number table. The treatment group codes so generated (A, B or C) were entered into cards along with the sample number, which were then placed in envelopes that were numbered and arranged sequentially. Allocation concealment was done by sealing the envelopes rendered opaque with aluminium foil. The envelopes were opened just before introducing the child to the behavioural intervention by the investigator.

### Blinding

Blinding of the patient, primary investigator and the assessors was not possible due to the nature of the intervention. However, the statistician who analysed the data was blinded to the group allocation.

### Statistical methods

All data were analysed using the SPSS (version 16.0) software package. The level of significance was set at 5% (i.e., *p* < 0.05). Mean values of pain, anxiety and behaviour measures were obtained for each group. Shapiro–Wilk test was used to assess normality of data. Kruskal–Wallis test was used for intergroup comparison of non-parametric data and one-way ANOVA was used for comparison of parametric data. Chi-square test was used for categorical variables. Paired t-test and Friedman test were used for within group comparison of dental anxiety values as measured by MCDAS _(f)_ scale and pulse rate respectively, at different time points. Multiple logistic regression was done with the type of distraction, along with factors that influence dental anxiety outcome such as socioeconomic status and gender of the child, parental anxiety, child temperament, past medical and dental experiences of the child as independent variables, to assess the effect of these factors as potential confounding factors on the dental anxiety outcome. Dental anxiety measured using MCDAS _(f)_ scale was the dependent variable.

## Results

**Fig. 2:**
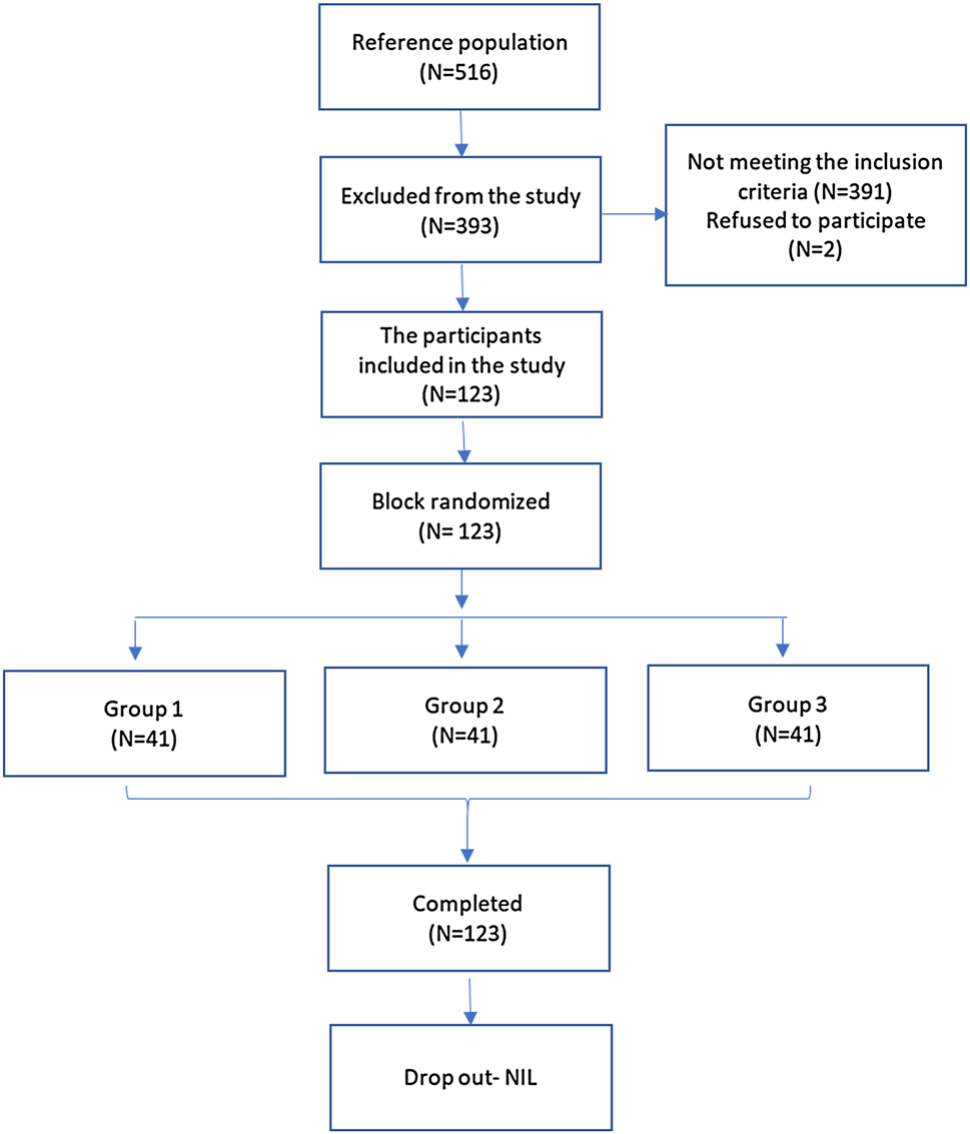
Flow chart showing the flow of participants in the study

Figure 2 illustrates the flow of participants through the study. There was no significant difference in the mean age and distribution of gender between the groups. There was a significant difference between the socioeconomic status of the groups, with the stress ball group showing a higher frequency of children belonging to upper socioeconomic status (Table 1).

**Table 1:** Descriptive statistics of the of the study population

Variable	Group 1 (stress ball)	Group 2 (audio-visual)	Group 3 (control)	*χ*^2^	*p* value
Age (mean ± SD)	9.90 ± 1.62	10.24 ± 1.65	9.87 ± 1.72	0.617^a^	0.541
Gender					
Male	25 (61%)	23 (56.1%)	20 (48.8%)	1.25	0.535
Female	16 (39%)	18 (43.9%)	21 (51.2%)		
Socioeconomic status					
Upper (16–25)	26 (63.4%)	12 (29.3%)	11 (26.8%)	14.31	0.001*
Lower (0–15)	15 (36.6%)	29 (70.7%)	30 (73.2%)		

*p < 0.05: significant

^a^*F* value

### Dental anxiety

**Fig. 3:**
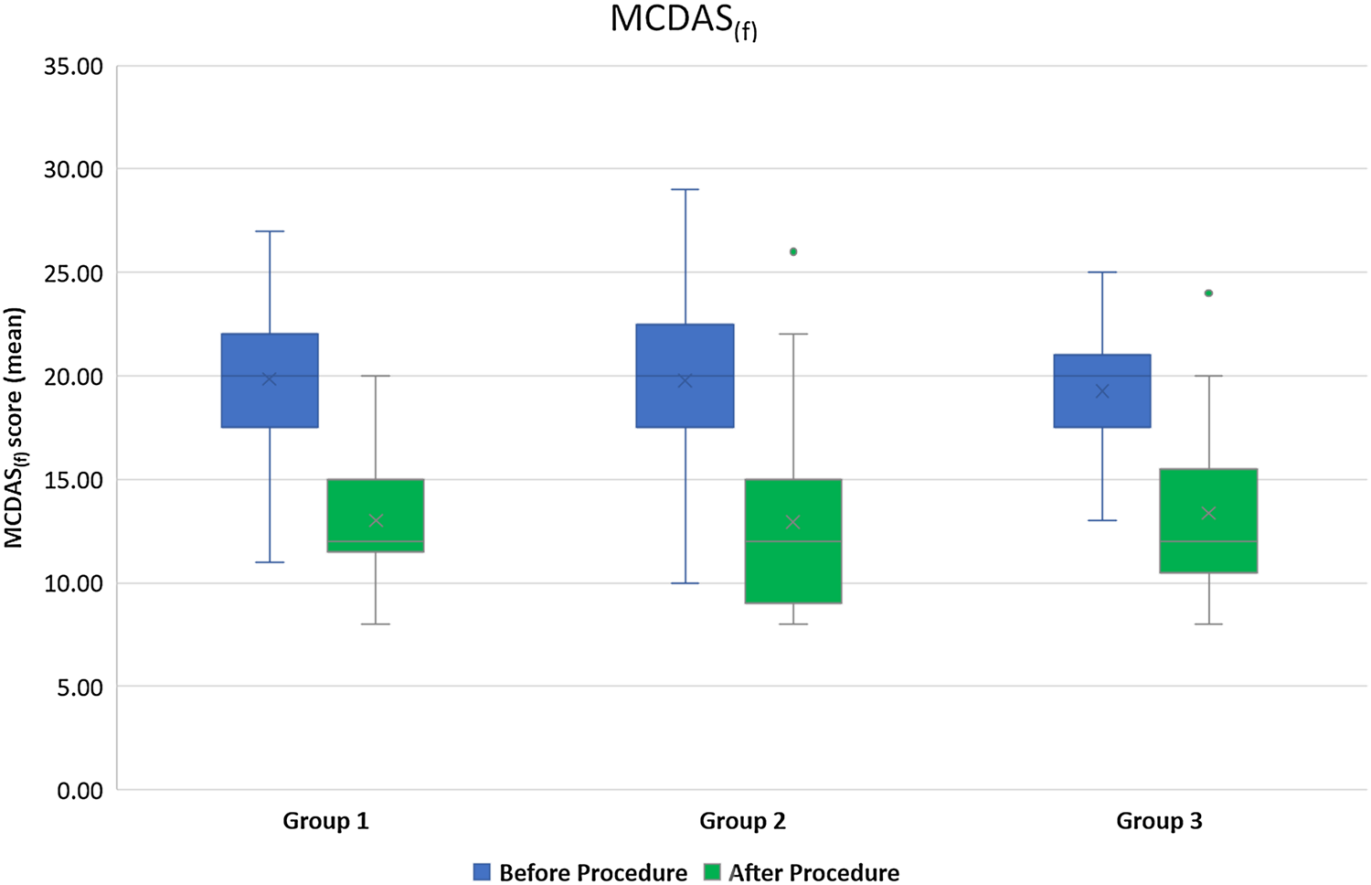
Mean MCDAS _(f)_ scores of the groups before and after the procedure

Among the eight questions of the MCDAS _(f)_ scale, in all groups, the most anxiety provoking question was having an injection in the gum; 46.3% of the participants “worried a lot” and 22.0% were “very worried” about having an injection, which decreased to 0.8 and 2.4%, respectively, after the intervention. In Group 2, 82.9% had mean scores < 19, signifying absence of state anxiety after the intervention, whereas it was 97.6% and 92.7% for Group 1 and 3, respectively. Intergroup comparison of the mean anxiety scores using one-way ANOVA did not yield a statistically significant value at both the time intervals (Before procedure: *F* = 0.14; *p* = 0.869; After procedure: *F* = 0.37; *p* = 0.688). Lower scores were observed for dental anxiety after the procedure than before, for all the three groups with the highest reduction seen in Group 2 (Fig. 3). Paired t-test showed a significant difference in the mean MCDAS _(f)_ scores obtained before and after the local anaesthesia administration, within all the groups (Group 1: *t* = 22.26; *p* < 0.001; Group 2: *t* = 14.33; *p* < 0.001 and Group 3: *t* = 10.91; *p* < 0.001).

Group 2 had the lowest pulse rate values, followed by Group 1, but Kruskal–Wallis test for intergroup comparison of the pulse rate values showed that the difference between the groups was non-significant (Table 2). The mean pulse rate showed variation with time and in all the groups, the highest values seen before the procedure, which increased during the procedure and decreased after procedure, resulting in mean pulse rates lower than the baseline. Group 3 had the highest pulse rates at all the time points (Table 2). Friedman test for within the group comparisons showed a significant difference in the pulse rate within the groups recorded at the three time points for Group 1 (*χ*^2^ = 35.73, *p* < 0.001), Group 2 (χ^2^ = 42.21, *p* < 0.001) and Group 3 (control group) (*χ*^2^ = 44.28, *p* < 0.001). Post hoc test (Bonferroni) showed a significant difference between before and during procedure, during and after the procedure mean pulse rate values in all the groups (Table 3). There was also a significant difference between the before and after mean pulse rate values in the control group.

**Table 2:** Comparison of the pulse rates between the groups

Group	Before		During		After	
	Mean (SD)	Range	Mean (SD)	Range	Mean (SD)	Range
Group 1 (stress ball)	101.75 ± 7.77	87–115	106.02 ± 5.11	99–115	100 ± 6.36	88–120
Group 2 (audio-visual)	99.95 ± 6.95	88–112	106.34 ± 6.73	99–129	99.48 ± 5.65	89–119
Group 3 (control)	103.56 ± 6.80	89–112	106.31 ± 6.13	99–125	100.26 ± 5.32	94–119
*H*	3.213		0.265		0.071	
*df*	2		2		2	
*P* value	0.201		0.876		0.965	

*SD* standard deviation

**Table 3:** Comparison of pulse rates within groups

Groups	Variable	Before vs during	During vs after	Before vs after
Group 1 (stress ball)	*Z*	3.13	4.43	1.53
	*p* value	0.002*	< 0.001*	0.125
Group 2 (audio-visual)	*Z*	4.73	5.62	1.25
	*p* value	< 0.001*	< 0.001*	0.212
Group 3 (control)	*Z*	2.97	5.62	3.64
	*p* value	0.003*	< 0.001*	< 0.001*

*p < 0.017: significant (Bonferroni correction)

### Behaviour

Non-parametric Kruskal–Wallis test showed no significant difference in the mean Venham’s behaviour rating scores between the groups (Table 4).

**Table 4:** Comparison of the Venham’s behaviour rating scores and Wong Baker Faces Pain Rating Scale (WBFPRS) scores for self-assessment of pain between the groups

Variable	Group	Mean ± SD	Range	*H* value	*p* value
Behaviour	Group 1 (stress ball)	0.76 ± 0.69	0.00–2.00	0.276	0.871
	Group 2 (audio-visual)	0.97 ± 1.08	0.00–4.00		
	Group 3 (control)	0.78 ± 0.76	0.00–3.00		
Pain	Group 1 (stress ball)	1.75 ± 1.15	0.00–4.00	5.26	0.072
	Group 2 (audio-visual)	2.12 ± 1.51	0.00–5.00		
	Group 3 (control)	2.36 ± 1.27	0.00–5.00		

*SD* standard deviation

### Pain assessment

The mean pain scores using self-reported WBFPRS scale were lower in Group 1 compared to Groups 2 and 3. Non-parametric Kruskal–Wallis test showed no statistically significant difference in pain scores between the groups (Table 4).

For intergroup comparison of the frequencies of pain score categories obtained using FLACC scale assessed by the observer, chi-square test was used. The results showed no significant difference in pain category frequencies between the groups (*χ*^2^ = 7.41; *p* = 0.284). Most of the participants in Group 1 and Group 2 showed mild discomfort (39% and 26.8%, respectively) or moderate pain (both 36.6%) while in Group 3 (control group), most showed mild discomfort (39%) or were relaxed and comfortable (29.3%).

### Co-variables

#### Temperament scores

Intergroup comparison of the mean values of emotionality, activity and shyness temperaments between the groups using one-way ANOVA showed a significant difference in activity and emotionality temperament between the groups. Group 1 showed higher activity and emotionality mean score followed by Group 2 (Table 5). Post hoc Tukey test showed that the mean activity temperament scores of the control group (Group 3) differed significantly from both Group 1and Group 2 (*p* = 0.001 and *p* = 0.002, respectively). A significant difference was noted between Groups 1 and 3 for mean emotionality temperament scores (*p* = 0.029).

**Table 5:** Comparison of temperament and MDAS scores between the groups

Variable	Group 1 (stress ball)	Group 2 (audio-visual)	Group 3 (control)	Test value	*p* value
	Mean ± SD	Mean ± SD	Mean ± SD		
Temperament (EAS)					
Emotionality	16.70 ± 2.41	15.29 ± 3.14	14.97 ± 3.40	3.83^a^	0.024*
Activity	11.85 ± 2.48	11.78 ± 2.61	9.97 ± 2.03	8.12^a^	< 0.001*
Shyness	18.04 ± 1.92	17.68 ± 2.36	17.80 ± 2.34	0.290^a^	0.749
Parental anxiety (MDAS)	8.63 ± 3.76	8.36 ± 3.08	7.75 ± 2.24	3.17 ^b^	0.205

*SD* standard deviation

*p < 0.05: significant

^a^*F* value

^b^*H* value

#### Parental anxiety

Majority of the parents were more anxious regarding ‘getting their teeth scaled and polished’ and while ‘sitting in the waiting room,’ when parental anxiety was assessed using MDAS scale. Intergroup comparisons using the non-parametric Kruskal–Wallis test showed no statistically significant difference in the parental anxiety (*p* = 0.423) (Table 5).

#### Past visit to paediatrician and dentist

All patients had visited a paediatrician earlier and around 15% had undergone hospitalisation with no statistically significant difference between the groups (*χ*^2^ = 2.37; *p* = 0.306). Most of the parents reported that the earlier visits to the paediatrician were perceived to be pleasant (very much = 40% and mostly = 60% on a Likert scale) by the child with no statistically significant difference between the groups (*χ*^2^ = 2.51; *p* = 0.285). Most of them had not visited a dentist earlier (87.8%, 68.3% and 92.7% in Groups 1, 2 and 3, respectively), with a significant difference between the groups (*χ*^2^ = 9.64; *p* = 0.008). Among those who visited, 97% of the participants perceived their earlier dental visits as pleasant, with no significant difference between the groups (*χ*^2^ = 3.05; *p* = 0.549).

None of the independent variables, such as gender, socioeconomic status, temperament, maternal anxiety, prior visit to paediatrician or dentist influenced self-reported dental anxiety outcome. Adjusted odds ratios showed no significant effect of active or passive distraction in decreasing self-reported dental anxiety (Supplemental Table 1).

## Discussion

In this study, the effect of active and passive distraction techniques during administration of local anaesthesia on dental anxiety (primary outcome), behaviour and pain levels (secondary outcomes) were compared with conventional basic behaviour guidance technique without distraction, as control. The statistical test results showed no significant difference between the groups for these outcomes.

A decrease in MCDAS _(f)_ scores were seen after the intervention in all groups. MCDAS _(f)_ is a measure of state anxiety (Howard and Freeman 2007). Before the event, the state anxiety is anticipatory in nature, and the manifestation of the anxiety is due to uncertainty about a future threat and inability to mitigate or avoid it (American Academy of Pediatric Dentistry 2020). Behaviour guidance measures help to cope with dental anxiety (Appukuttan 2016; American Academy of Pediatric Dentistry 2020). As some form of behaviour guidance was used in all the groups, there was a significant decrease in self-reported state anxiety after the local anaesthesia procedure. The pulse rate showed variation with time that increased during the procedure and decreased after procedure. The pulse rate is governed by the autonomic nervous system, which reflects if the person is under stress or relaxation (Kreibig 2010). The anticipation of injection possibly provides sympathetic stimulation and catecholamine release, which accounts for an increase in pulse rate (Prabhakar et al. 2007).

The use of distraction techniques did not provide a significant advantage in decreasing anxiety and pain and improving behaviour when compared to the use of only conventional behaviour guidance techniques, such as effective communication and verbal positive reinforcement. AV eyeglasses engage the child’s attention due to both auditory and visual stimuli that activate cognitive and emotional centres of the nervous system, triggering positive emotions, resulting in a relaxed experience (Al-Khotani et al. 2016). However, there is a partial visual obstruction of the operating environment for the child, while allowing communication with the dentist (Ram et al. 2010). As the child is unable to view the real world around him, this may cause the fear of unknown; the surrounding unfamiliar environment may be perceived as threatening, leading to an increase in anxiety and pain perception (Al-Halabi et al. 2018). While audio-visual eyeglasses passively engage the child, the willingness, and the cognitive ability of the child to respond to commands and understand instructions in a scenario where the child is experiencing pain and distress, plays an important role in the effectiveness of active distraction technique like stress ball (Koller and Goldman 2012). This may have affected the effectiveness of the stress ball as a distracter by the child. Although there is limited data about the use of a stress ball during dental procedures, it has been recommended for use during paediatric procedures. While a stress ball is easy to use, inexpensive, considered interesting and entertaining for children (Sadeghi et al. 2013; Aydin et al. 2016), the need for maintenance of the AV glasses, the cost associated and the non-availability of sizes that accurately fit children with small faces are limitations of AV eyeglasses (Ram et al. 2010).

Communicative management and appropriate use of commands are applied universally in paediatric dentistry for both cooperative and uncooperative children. At the beginning of a dental appointment, asking questions and active/reflective listening can help establish rapport and trust and reduce anxiety (Appukuttan 2016). Communication methods such as ask-tell-ask allow the patients to ask questions about treatment and express their fears and anxiety. The ability of the dentist to listen to the patient’s concerns and build a two-way communication with empathy establishes rapport and acts as a trust-building measure, helping to decrease anxiety (American Academy of Pediatric Dentistry 2020). Positive reinforcement in the form of praise during the injection of the local anaesthetic acts as a positive feedback, increases the likelihood of recurrence of the behaviour and decreases anxiety (American Academy of Pediatric Dentistry 2020; Appukuttan 2016).

Earlier systematic reviews have found only a low level of evidence to conclude that distraction techniques are effective in mitigating dental anxiety (Prado et al. 2019; Liu et al. 2019). Studies have shown differences in results that are attributed to heterogeneity of outcome measures and variation in dental procedures studied (Liu et al. 2019). While some studies reported a decrease in dental anxiety and pain perception with improvement in behaviour, after use of distraction, the results are not consistent. Ram et al. (2010) investigated the effect of AV eyeglasses on the behaviour of children and observed it to be better than the control group (without the distraction aid). Nuvvula et al. (2015) showed that three-dimensional (3D) audio-visual distraction significantly reduced dental anxiety and improved behaviour in children aged 7–10 years, while Aminabadi et al. (2012) also showed that AV eyeglasses could decrease state anxiety and pain perception, among children aged 4–6 years, when used during restorative treatment. Another study comparing the active and passive distraction technique during local anaesthesia administration concluded that active and passive distraction techniques are comparable in reducing pain outcome (Abdelmoniem and Mahmoud 2016). However, Al-Halabi et al. (2018) saw no significant difference after the use of virtual reality eyeglasses in self-reported pain perception measured using WBFPRS and FLACC scale in a study. In another study, improvement in behaviour among 6–8-year-old children was seen after use of audio-visual distraction, although no decrease in anxiety and pain perception was seen (Guinot Jimeno et al. 2014). Al Khotani et al. (2016) reported a decrease in observer rated anxiety and improvement in behaviour among children using AV eyeglasses as a distraction as compared to control group without distraction, though no difference was seen in self-reported anxiety. The results of our study are in contrast to results obtained with the use of a stress ball during paediatric intervention techniques. Aydin et al. (2016) and Aykanat Girgin and Göl (2020) found that use of a stress ball was effective in decreasing pain and anxiety when used during phlebotomy and venipuncture, respectively, in 7- to 12-year age group.

Hoge et al. (2012) in their study did not find a significant difference in pain perception after use of AV glasses but found a decrease in disruptive behaviour. The decrease was more pronounced in patients below 8 years of age and was not significant for patients above 8 years of age. The sample used in our study consisted of patients above 8 years of age who showed positive behaviour. Thus, it may be inferred that among 8–12-year-old childrern with Frankl’s positive behaviour, use of stress ball or AV eyeglasses as distraction technique does not provide additional benefit over the use of conventional basic behaviour guidance techniques (like communication and verbal positive reinforcement) only. However, the results of the study do not contradict the use of active or passive distraction as a behaviour guidance technique by paediatric dentists.

In our study, care was taken to conduct the intervention with standardized procedures by a trained single operator and the observer outcomes were measured using a video of the intervention, calibrating for reliability of the assessment. The developmental, cognitive and situational factors may influence the child's perception of anxiety and pain and thus influence the outcomes of self-reported scales such as MCDAS _(f)_ and WBFPRS (Stinson et al., 2006). Thus, objective parameters like pulse rate which is a reflection of the physiological changes that occur in the body in response to stress and anxiety during dental treatment (Guinot Jimeno et al. 2011) and observational pain scales like FLACC were used as adjunct measures for anxiety and pain assessment, respectively.

However, our study has a few limitations. Blinding of assessor or the participant was not possible due to the nature of the intervention. Although earlier studies (Ram et al. 2010; Hoge et al. 2012; Nuvvula et al. 2015; Aydin et al. 2016) have shown good acceptability of both AV eyeglasses and stress ball techniques by both parents and children, the qualitative data regarding acceptability and comfort were not obtained in our study. Parallel design was chosen over cross over design for the study, to avoid spillover effect due to the dental experience during the earlier appointments. (Ram et al. 2010). However, a parallel study design may result in inter-participant variability between the groups, which can be a limitation (Aminabadi et al. 2012).

To minimize confounding due to inter-participant differences in co-variables that can affect the dental anxiety (primary outcome), a multiple logistic regression analysis including co-variables was done that showed no significant differences (Howards 2018). Future studies can explore the effect of AV eyeglasses and stress ball distraction techniques during other dental treatment procedures, the effectiveness of distraction techniques over consecutive treatment appointments and involving children with Frankl’s negative behaviour.

## Conclusion

Considering the limitations of the present study, it appears that for children in the age group 8–12 years, with Frankl’s positive behaviour in the dental clinic, the use of conventional basic behaviour guidance with or without distraction was effective in reducing dental anxiety. The use of active stress ball distraction or passive AV eyeglass distraction during administration of local anaesthesia does not significantly improve behaviour and reduce dental anxiety and pain levels, as compared to the use of conventional basic behaviour guidance methods such as effective communication with euphemisms and verbal positive reinforcement, without distraction.

## Supplementary Information

Below is the link to the electronic supplementary material.

Supplementary file1 (DOCX 16 KB)

## Data Availability

Data are available from corresponding author on request.
