# Supplementary material for: Effect of active and passive distraction techniques while administering local anaesthesia on the dental anxiety, behaviour and pain levels of children: a randomised controlled trial
Source: Eur Arch Paediatr Dent. 2022 Mar 10;23(3):417–27. doi: 10.1007/s40368-022-00698-7 (PMC9167192; doi:10.1007/s40368-022-00698-7)
Supplement: Supplementary file 1 — Supplementary file1 (DOCX 16 KB) [file 40368_2022_698_MOESM1_ESM.docx]

**Supplemental Table 1: Multiple logistic regression model with presence of state anxiety (MCDAS _(f)_ ≥19) as dependent variable**

| **Variable** |  | **Wald** | **p- value** | **Odds ratio** | **Confidence Interval**  **(Lower-Upper)** |
| --- | --- | --- | --- | --- | --- |
| **Gender** | **Male** | 1.60 | 0.205 | 3.3 | 0.52-20.81 |
|  | **Female** |  |  |  |  |
| **Socioeconomic Status** | **Upper** | 1.26 | 1.478 | 0.22 | 0.037-2.16 |
|  | **Lower** |  |  |  |  |
| **Mean MDAS score** |  | 1.53 | 0.217 | 1.15 | 0.92-1.42 |
| **Temperament** | **Emotion** | 0.67 | 0.412 | 0.88 | 0.65-1.19 |
|  | **Activity** | 1.42 | 0.234 | 0.67 | 0.38-1.26 |
|  | **Shyness** | 1.37 | 0.241 | 1.23 | 0.87-1.72 |
| **Visit to Paediatrician pleasant** | **Yes** | 1.50 | 0.221 | 3.46 | 0.47-25.33 |
|  | **No** |  |  |  |  |
| **Visited a dentist before** | **Yes** | 2.77 | 0.096 | 0.16 | 0.02-1.39 |
|  | **No** |  |  |  |  |
| **Group 1** |  | 0.02 | 0.877 | 0.78 | 0.03-18.89 |
| **Group 2** |  | 2.19 | 0.139 | 4.78 | 0.60-37.91 |
